# The ethics of research into health and climate change: call for papers

**DOI:** 10.2471/BLT.24.292990

**Published:** 2025-01-01

**Authors:** Katherine Littler, Julian Sheather, Adrienne Sayer

**Affiliations:** a Health Ethics and Governance Unit, Research for Health Department, Science Division, World Health Organization, Avenue Appia 20, 1211 Geneva 27, Switzerland.; b Geneva, Switzerland.

Climate change is the greatest health threat facing humanity.^1^^–^^5^ The nature and scale of the interconnected impacts of climate change on health as well as the effectiveness of interventions to adapt to and mitigate climate change are currently the focus of research.

Climate and health research must address questions of justice and fairness while balancing the need to act with caution amid uncertainties, climate complexities and methodological limitations of current methods and metrics. Responsibility for climate change and historical anthropogenic emissions lies overwhelmingly with industrialized countries, but the health impacts of climate change fall disproportionately on people in settings with the least resources and capacity to mitigate and adapt to climate change.

Thus, in addition to fulfilling the usual ethical requirements of research, research on the health impacts of climate change must address additional ethical questions and concerns. Climate and health research frequently engages with disadvantaged populations affected by climate change or disasters,^6^ who are unlikely to benefit directly from this research. Studies investigating the nature and magnitude of health harms resulting from climate change can raise questions about how to communicate the risks, especially when few ways exist to alter them locally. Other challenges include safeguarding research participants against exploitative research practices, ensuring well-being and respect for participants, achieving meaningful participation and co-creation, and identifying what ancillary care or complementary services might be appropriate.

While climate emergency responses that draw on Indigenous Knowledges and One Health and Planetary Health approaches^7^^–^^9^ recognize the value in non-human aspects of the biosphere, scientific health research focuses primarily on human-centred benefits. Climate change challenges these anthropocentric norms. The scope of ethical considerations of climate and health research may therefore need to be expanded to include more-than-human interests, acknowledge the value of ecosystems, and consider possible trade-offs between human and non-human health in research. Health concepts that provide a normative basis for climate and health research must also ensure they are inclusive of divergent worldviews, including Indigenous concepts of health that may not be distinct from concepts of land, place or environment.

Action on climate change frequently benefits health, often bringing matching, if not fully measured, economic gains. Reductions in fossil fuel use, for example, can reduce air pollution and associated deaths attributable to air pollution, bringing potentially considerable economic benefits. Research into the health impacts of climate change can, however, highlight challenging compromises between health and short-term economic gains as well as health–health trade-offs, where choices have to be made between different kinds of health benefits, or that favour health benefits for some populations over others.^10^ Climate and health research also raises questions about global and intergenerational justice,^11^ particularly when investigating interventions with uneven benefits and harms across regions and timeframes. The presence of these trade-offs and their implications for transgenerational equity^12^ require consideration to mitigate potential harms, underlining the centrality of ethics in research on climate change and health.

Questions about which research should be given priority are critical, and ethical considerations are underdeveloped. To address this gap, the *Bulletin of the World Health Organization* will publish a theme issue on the ethics of research into health and climate change. This issue will investigate a range of topics shaping ethically sound climate and health research across a range of methods, populations and disciplines. Papers considered for the theme issue need to provide an advance in the field, and should respond to critical questions such as: what should be investigated and why? What should take priority and according to which criteria? Who or which entities should make decisions regarding the research agenda and by which process(es)? Given that some countries and populations are facing existential threats from climate change – including the risk that their homelands will cease to exist within decades – what does fair and ethical research into the effects of climate change on their health require? Additionally, the theme issue will cover ethical aspects of climate- and health-related research as they pertain to policy.

The *Bulletin* welcomes contributions from all stakeholders including public health decision-makers, researchers, civil society and community representatives by 1 June 2025. Manuscripts should be submitted in accordance with the *Bulletin’s* guidelines for contributors (available at: https://www.who.int/publications/journals/bulletin/contributors/guidelines-for-contributors) and the cover letter should mention this call for papers. 
